# Editorial: Molecular mechanisms and treatment of MYCN-driven tumors, volume II

**DOI:** 10.3389/fonc.2025.1690803

**Published:** 2025-09-05

**Authors:** Yuyan Zhu, Yusuke Suenaga

**Affiliations:** ^1^ Department of Urology, The First Hospital of China Medical University, Shenyang, China; ^2^ Laboratory of Evolutionary Oncology, Chiba Cancer Center Research Institute, Chiba, Japan

**Keywords:** MYCN, hepatocellular carcinoma, medulloblastoma, therapeutic targets, oncogene

Series of papers on this topic focuses on the central role of MYCN (N-myc proto-oncogene protein) in tumorigenesis and its targeting strategies. As a key member of the MYC family, the amplification and overexpression of MYCN are widely observed in highly aggressive tumors such as neuroblastoma, hepatocellular carcinoma (HCC), and medulloblastoma, and are significantly associated with poor prognosis. By integrating multidisciplinary research findings, series of papers systematically elucidates the pathological mechanisms of MYCN in tumorigenesis and explores potential innovative therapeutic strategies.

MYCN is a member of the MYC family of transcription factors and plays a critical role in neural development by regulating cell proliferation, differentiation, and apoptosis. Abnormal expression of MYCN through amplification or mutation (e.g., P44L, T58M) has been found by Fernandez Garcia et al. to contribute to various pediatric brain tumors—such as neuroblastoma, medulloblastoma, and high-grade gliomas—as well as embryonal tumors, and is associated with poor prognosis. Nishio et al., summarizing previous studies, reported that dysregulation of MYCN function during development can also lead to genetic disorders: loss of function causes Feingold syndrome (characterized by microcephaly and digit anomalies), while gain of function leads to megalencephaly-polydactyly syndrome. Strategies targeting MYCN include inhibiting its transcription (using BET inhibitors), promoting its degradation (e.g., with olaparib), or blocking downstream pathways (such as with CDK7 inhibitors). However, challenges such as the blood-brain barrier and the “undruggable” nature of the protein remain.

Xu et al. established a high-throughput screening platform based on a MYCN promoter luciferase reporter system. Using this model, they screened 9,600 compounds and ultimately identified MI202 as a compound that inhibits MYCN transcriptional activity and gene expression in a dose-dependent manner. This compound specifically suppressed proliferation and colony formation in liver cancer cells and induced apoptosis, while exhibiting no significant toxicity in normal hepatocytes. Through genome-wide CRISPR-Cas9 screening, Xu et al. further identified ACOT2, a key molecule in lipid metabolism, as the functional target of MI202. Downregulation of ACOT2 mediated the inhibitory effect of MI202 on MYCN and contributed to its pro-apoptotic activity. This study demonstrates that MI202 suppresses hepatocellular carcinoma progression by targeting the ACOT2–MYCN pathway, providing a new candidate compound and a potential therapeutic target for targeted liver cancer therapy.

Zwaig et al. applied 10x Genomics linked-read sequencing technology to perform whole-genome analysis on 25 medulloblastoma cases (primarily Group 4), aiming to decipher the complexity of structural variations and point mutations. The study successfully detected a variety of rare and complex genetic events, including enhancer hijacking, extrachromosomal DNA (ecDNA) harboring MYCN, chromothripsis, and complex chromosomal interactions, and provided the first confirmation that linked-read technology can be used for ecDNA detection. The research also validated that PRDM6 overexpression occurs through mechanisms other than SNCAIP duplication and identified several germline mutations associated with DNA repair pathways. The results demonstrate that linked-read technology enables comprehensive and efficient identification of complex variants in medulloblastoma with low sample input, revealing the high heterogeneity of the Group 4 subtype and providing new insights into its molecular mechanisms and clinical diagnosis.

This topic integrates basic mechanistic insights and translational research to systematically outline multi-dimensional strategies for targeting the “undruggable” MYCN oncoprotein. From upstream transcriptional regulation and protein stability maintenance to downstream metabolic reprogramming, each node offers potential therapeutic opportunities. Future studies should focus on developing tissue-specific delivery tools, elucidating resistance mechanisms, exploring the remodeling of the immunometabolic microenvironment, and promoting cross-cancer clinical trials of combination therapies, ultimately translating these scientific discoveries into precision medicine strategies that can benefit patients.

